# A Microfluidic Bioreactor with *in Situ* SERS Imaging for the Study of Controlled Flow Patterns of Biofilm Precursor Materials

**DOI:** 10.3390/s131114714

**Published:** 2013-10-29

**Authors:** François Paquet-Mercier, Nahid Babaei Aznaveh, Muhammad Safdar, Jesse Greener

**Affiliations:** Département de Chimie, Université Laval, 1045 Avenue de la Médecine, Québec, QC G1V 0A6, Canada; E-Mails: francois.paquet-mercier.1@ulaval.ca (F.P.-M.); nahid.babaei-aznaveh.1@ulaval.ca (N.B.A.); muhammad.safdar.1@ulaval.ca (M.S.)

**Keywords:** Surface Enhanced Raman Spectroscopy, spectral imaging, microfluidic, biofilm, electroless deposition, bioreactor, plasmonic surfaces

## Abstract

A microfluidic bioreactor with an easy to fabricate nano-plasmonic surface is demonstrated for studies of biofilms and their precursor materials *via* Surface Enhanced Raman Spectroscopy (SERS). The system uses a novel design to induce sheath flow confinement of a sodium citrate biofilm precursor stream against the SERS imaging surface to measure spatial variations in the concentration profile. The unoptimised SERS enhancement was approximately 2.5 × 10^4^, thereby improving data acquisition time, reducing laser power requirements and enabling a citrate detection limit of 0.1 mM, which was well below the concentrations used in biofilm nutrient solutions. The flow confinement was observed by both optical microscopy and SERS imaging with good complementarity. We demonstrate the new bioreactor by growing flow-templated biofilms on the microchannel wall. This work opens the way for *in situ* spectral imaging of biofilms and their biochemical environment under dynamic flow conditions.

## 1. Introduction

Microfluidic (MF) technology is finding new opportunities in biomaterial synthesis due to exceptional control of reaction variables, control over mixing, reduction of material consumption, and isolation of the growth environment from ambient conditions. In addition, the well-established fabrication of MF devices opens the way for rapid optimization of complex channel designs [1,2] achieving desired flow properties, precise timing between stages of material synthesis [3], parallelisation [4], and integration of functional components such as electrodes, valves, and temperature control elements [5–7]. The use of MFs for studies of biofilms is very attractive due to the ability to precisely control the wall shear stress, temperature and chemical gradients. Generally, *in situ* imaging of biofilms and other biomaterials is accomplished by 2D or confocal microscopy techniques [8–10]. However, the rapid pace of MF-based material development has placed strong demands on *in situ* characterization [11]. To this end, there has been increasing demand for new spectral imaging methods that can simultaneously report on a broad range of chemical properties without needing foreign probe molecules. Elegant approaches to spectral imaging, include those based on attenuated total reflection infrared (ATR-IR) spectroscopy, synchrotron-radiation based infrared (SR-IR) spectroscopy and magnetic resonance imaging (MRI) [12–16]. These approaches are promising, but are not widely utilized due to the complexities in their setup and their interface with the MF environment, cost, and challenges in time of acquisition, spatial resolution and sensitivity. Raman spectral imaging solves some of these problems. Photons from laser excitation and inelastic scattering are in the visible spectrum, which are not absorbed strongly by most common MF fabrication materials or by aqueous environments. Raman also supports excellent spectral resolution, a wide spectral window and has very good diffraction-limited spatial resolution on the order of microns supporting studies of mass transport, chemical reactions and material synthesis [17–19]. Reaction kinetics and other quantitative studies can be conducted by Raman using the linear relation between scattering intensity and analyte concentration given by:

(1) $$I_{R}\propto \sigma \cdot \nu^{4}\cdot P\cdot t\cdot C$$

where *I_R_* is Raman scattering intensity, σ is the inelastic scattering cross-section, *v* is excitation laser frequency, *P* is the laser power, *t* is acquisition time and *C* is analyte concentration. However, one of the major drawbacks of Raman spectroscopy is its low sensitivity due to values of σ that are on the order of 10^−30^ to 10^−25^ cm^2^. This is especially challenging for sensing biofilms and their precursor materials due to their low densities and concentrations, respectively. Attempts to overcome this problem by increasing *v*, *P*, or *t* in Equation (1) can result in sample heating or thermal breakdown of analytes, whereas long acquisition times are not desirable, nor feasible for biomaterials that change their morphology relatively rapidly. In addition, high frequency photons can induce fluorescence in organic molecules and polymer MF fabrication materials.

Surface Enhanced Raman Spectroscopy (SERS) is a useful approach that takes advantage of plasmonic properties of metal nanostructures to increase Raman scattering intensity by orders of magnitude due to strong increases in local electric fields between metal nanostructures. Fluorescence quenching, which is known to occur at the surfaces of many metals used for SERS, is an additional benefit. To date, most studies using SERS in MF environments, including those focusing on biomaterials, have utilised suspended gold or silver nanoparticles [20–24]. However, there are several drawbacks to this approach. First, signal instability due to differential SERS enhancement in time and space can undermine the goal of quantitative spectral imaging. For example, continuous processes, such as nanoparticle build-up on channel walls, sedimentation and other so-called memory effects, can slowly change SERS enhancement [25,26]. In addition, spatial uniformity requires strong mixing of nanoparticle colloids. Aggregation in salt-containing growth solutions can destabilize nanoparticles colloids, which rely on electrostatic repulsion. Second, nanoparticles are lost in MF reactors due to washout. Lastly, SERS measurements at the MF channel wall, where biomaterials tend to grow, is difficult using nanoparticle colloids because the majority of the signal comes from the bulk liquid phase. As an alternative, the addition of solid nanostructured metal SERS surfaces to microchannel walls is attractive because they are stable and enhancement is localised where biomaterial deposition and growth occurs. Photo induced nanostructuring can be achieved by photoreduction of silver precursors or fast thermal annealing of thin gold films to obtain nanostructured silver [27,28] and gold [29] SERS surfaces, respectively. However, these techniques require femtosecond laser pulsing or strong UV lasers. The latter can cause heat damage and photo-breakdown of MF fabrication materials. In another approach, metal is deposited over nanostructured substrate within the microchannel, but this requires complex lithographic steps in advance of metal deposition [30–34].

In response to the need for new, user-friendly fabrication of SERS for spectral imaging in MFs, we present a MF bioreactor with the following functionalities: (1) strong signal enhancement for rapid, low-background measurements of mM biofilm precursor solutions using a low-power laser; (2) the ability for localized deposition of biofilm precursor materials and flow-templated biofilm growth on a single MF wall; (3) the ability for unobstructed optical and spectroscopic imaging of the entire cultivation surface. The system was demonstrated by generating one-dimensional spectroscopic images of the local concentration of a spatially confined precursor solution and compared to optical micrographs of the flow stream and flow-templated biofilms cultivated under the same flow conditions.

## 2. Experimental Design and Methods

Microfluidic bioreactors were made from polydimethylsiloxane (PDMS, Sylgard 184, Dow Corning, Midland, MI, USA). Bonding PDMS to secondary PDMS levels or to glass coverslips was accomplished by exposing bonding surfaces to air plasma for 90s using a plasma cleaner (PCD-001 Harrick Plasma, Ithaca, NY, USA) operated at 600 mTorr at a power of 29.6 W. Nanostructuring of metal layers was achieved by exposure to air plasma from the same plasma system and settings, but for different times.

Liquids were introduced into the MF bioreactor *via* syringe pumps (PHD 2000, Harvard Apparatus, Holliston, MA, USA). All liquids were first degassed in order to prevent bubble formation. Chemicals used for electroless deposition of metal layers included silver nitrate, l-tartaric acid, glucose, gold(III) chloride and sodium bicarbonate (Sigma Aldrich, Saint-Louis, MO, USA). Sodium citrate was provided by Sigma Aldrich. Ultrapure water with a resistivity of 18.1 MΩ·cm^−1^ was used for all solutions. Due to short shelf life, Tollens reagents were made fresh by adding ammonium hydroxide to AgO_2_ precipitate prepared by mixing silver nitrate solution with sodium hydroxide solution until dissolution. Food colours were used for visualisation of the flow (McCormick, London, ON, Canada).

Atomic force microscopy (AFM, Nanoscope III Multimode, Digital Instruments, Santa Barbara, CA, USA) was used to perform topographic analysis of the silver SERS layer. The AFM measurements were conducted in tapping mode at ambient conditions. A J-scanner was used with NSC15\AlBS silicon standard probes (Mikromasch, Lady's Island, SC, USA). The silver layer was deposited following the same protocol as adopted for the preparation of SERS active microfluidic channels. Each measurement was performed on a total scan area of 100 μm^2^. Scan rate was 0.25 Hz and the amplitude set point was between 1.3 V and 1.6 V. Height, amplitude and phase images were collected simultaneously. Data acquisition and roughness analysis was performed using the Nanoscope software version 5.30r3.

Diffuse reflectance UV-Vis spectra were recorded using a Cary 500 Scan spectrophotometer (Varian, Palo Alto, CA, USA) with a Praying Mantis™ diffuse reflectance accessory (Harrick Scientific, Pleasantville, NY, USA).

Raman spectra were recorded using a LABRAM 800HR Raman spectrometer (Horiba JobinYvon, Villeneuve d'Ascq, France) coupled with an Olympus BXmicroscope with a 100× long focal objective (NA 0.75) in backscattering mode. An Ar^+^ laser (Coherent, INOVA 70C Series Ion Laser, Santa Clara, CA, USA) provided the excitation source *v* = 514.5 nm. Measurements were conducted with a 200 μm slit and 100 μm confocal hole. For SERS measurements, laser power was reduced from 100 mW to 10 mW using a neutral filter with an optical density of 1. The full spectra were acquired in three spectral windows for total acquisition time of one minute. Optical micrographs were recorded using an Axioskop microscope (Zeiss, Jena, Germany) with an external light source (Illuminator, Cole-Parmer Canada, Montreal, QC, Canada). A home-built polycarbonate holder was used to accommodate the fluidic connections and achieve the proper orientation for Raman and optical inspection.

Raman and UV-Vis spectra were treated and analysed using Grams/AI 8.0 for baseline correction, peak deconvolution and intensity measurements. Optical density data were extracted from micrographs using the open source software ImageJ V1.47.

For descriptions of processes related to bacterial culture, system sterilization, inoculation and biofilm culturing, readers are referred to the section on biological materials preparation in the Supplementary Materials of this paper.

### 2.1. Fabrication of a Two-Level Bioreactor for Flow Confinement against the SERS Surface

**Figure 1.**
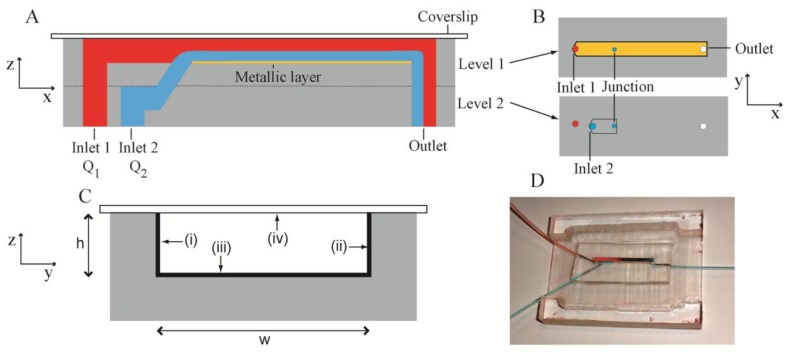
(**A**) Schematic showing the cross-section of a two-level MF bioreactor in the x-z plane. Inlet 1 is used to introduce the sheath flow solution (red) at flow rate Q_1_. Inlet 2 is used to introduce the biofilm precursor flow (blue) at flow rate Q_2_. The red flow confines the blue flow at the bottom of the microchannel. (**B**) Schematic of the two levels in the x-y plane. The junction, the inlets and the outlet are represented by circles. (**C**) Schematic showing the cross-section of the Level 1 channel in the y-z plane. The channel walls consist of PDMS (grey) covered with an opaque metal layer (black) on the side walls (i, ii) and the bottom SERS sensing surface (iii). A glass sealing layer defines the fourth channel surface (iv). The channel dimensions are h = 305 μm and w = 2 mm (not to scale). (**D**) Photograph of an operational MF bioreactor with inlet tubes delivering red and blue coloured solutions to inlets 1 and 2, respectively. The MF bioreactor is oriented with the glass side up for inspection by Raman spectrometer and optical imaging. The holder provides space on the PDMS side of the bioreactor for tubing to be connected to inlets and outlet via metal elbow joints.

The present microbioreactor was a two-level system (Figure 1A) fabricated in PDMS. The channel structures were fabricated by casting uncrosslinked PDMS against a silicon mould with patterned photoresist features. These features had the inverse dimensions of the required channels, but resulted in the required channel dimensions in the PDMS following casting. Levels 1 and 2 consisted of channels with dimensions of width w = 2 mm, height h = 305 μm and length l_1_ = 32 mm and l_2_ = 9 mm, respectively (Figure 1B). The two levels were aligned and bonded such that the channels therein were collinear and there was overlap between them. A cylindrical junction was formed between the overlapping segments using a punch (diameter = 500 μm). The punch angle was 45 degrees, such that the liquid entering the channel in Level 1 had some component of its velocity in the x-direction in order to: (i) keep the biofilm precursor stream close to the bottom of the Level 1 channel; (ii) reduce shear forces between the two streams and (iii) maintain smooth laminar flow. Level 1 channel was sealed by a glass cover slip with thickness of 170 μm, which matched the working distance of the Raman spectrometer system. Confining liquid (pure water) and biofilm precursor liquids (bacterial inoculants and citrate solutions) were introduced into Level 1 and Level 2 channels *via* Inlet 1 and Inlet 2, with a flow rate Q_1_ and Q_2_, respectively. Figure 1C shows a cross-section of the sealed Level 1 channel with three metalized channel walls and the glass sealing layer. Characterization took place against the bottom surface in Level 1. The entire assembled bioreactor is shown in Figure 1D.

### 2.2. Electroless Metal Deposition on Microchannel Walls

The bottom and side walls of the of the Level 1 channel were covered with a metallic layer *via* electroless deposition [35]. Unlike electrodeposition, this approach enabled deposition against non-conducting PDMS microchannel surface. Electroless deposition of a silver layer was achieved by combining an aqueous solution of glucose, tartaric acid and ethanol with a Tollens reagent. The bioreactor was masked using an adhesive film (HDClear, Henkel Corp., Düsseldorf, Germany) such that only the channel section was exposed. Before deposition, the microchannels were treated by air plasma at 600 mTorr at 29.6 W for 90 s in order to increase their hydrophilicity which allowed a better wetting by the aqueous solution. After the reaction was complete, the excess solution was removed and the channel was washed with ultrapure water and dried with filtered nitrogen. After deposition, the bottom and side walls of the channel were coated by a matt grey silver film. This conductive layer had a resistivity of 110 Ω/m. The mask, which protected the bonding surfaces, was then removed leaving silver in the channel only. Gold layers were formed in a similar way, but the results are not reported here because further optimisation is required to improve their SERS enhancement.

### 2.3. Transformation of the Metal Surface to a Sensitive SERS Surface for Spectral Imaging

After metal deposition, a weak signal enhancement was observed, presumably due to the slightly roughened surface after the Tollens reaction. Nevertheless, further enhancement was required to observe low citrate concentration solutions in biofilm growth media. This was accomplished by exposing the metal surface to air plasma, which enhanced nanostructuring and helped clean residual organic impurities left over from the electroless deposition process *via* sputtering and oxidation [36–38]. Nanostructuring, plasmonic enhancement, and resulting SERS were observed after different plasma exposure times by atomic force microscopy (AFM), UV-Vis and Raman spectroscopy, respectively. As shown in Figure S1, AFM images of the plasma treated metal surfaces showed an initial rapid increase followed by a plateau after nearly 20 min exposure. Over this time frame, the total increase in mean surface roughness (Ra) was over 40%, as expected [39,40]. The resulting plasmonic absorption bands were observed from UV-Vis spectra collected in diffuse spectral reflectance mode. The band increased in both magnitude and width with plasma exposure time.

**Figure 2.**
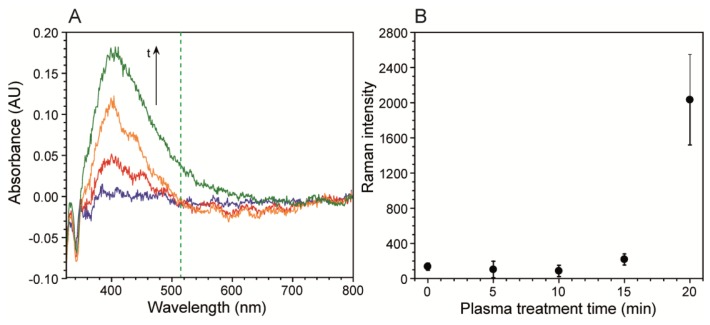
(**A**) UV-Vis spectra of the silver surface after 1 (blue), 5 (red), 10 (orange) and 19 (green) minutes of plasma treatment. Spectra were acquired in diffuse reflectance mode with an integration time of 0.6 s and 1nm data intervals. Each absorbance spectrum in (A) was generated using the spectrum of the silver surface after 0 min plasma treatment as the background spectrum. The green broken line at 514.5 nm in (A) represent the wavelength of the excitation laser source; (**B**) Raman intensity for the νC-COO band of sodium citrate at 952 cm^−1^ with increasing plasma treatment times using a 514.5 nm excitation laser. Air plasma treatments were done at power of 29.6 W at pressure of 600 mTorr. Error bars were generated for separate measurement at different locations on the same substrate.

As seen in Figure 2A the band is near 400 nm, as expected for silver. Figure S2 shows the results of the mathematical deconvolution of this peak into three distinct absorption bands, which included the main band at 397 nm, and two smaller bands at 439 nm and 466 nm. Figure S2 shows the evolution of the individual band intensities with plasma exposure time, which featured a high initial slope followed by a plateauing after long exposure times, resembling the Ra response. The band broadening occurred on the low frequency side, due to the growth of two secondary bands. It was primarily the low frequency tail of the 466 nm band, which overlapped with the 514.5 nm Raman excitation source. We followed the resulting SERS intensity of a 5 mM citrate solution after five different plasma treatment times. Figure 2B shows that intensity of the νC-COO citrate band was nearly unchanged at exposure times of 15 min or less, but increased after that. This corresponded to the spectral density increases at the Raman laser excitation frequency shown in Figure 2A. Future optimisation should include longer plasma exposure times, to maximize Ra, and the use of higher frequency excitation lasers, to achieve better overlap with the silver absorption band.

## 3. Results and Discussion

### 3.1. Eliminating Background Signals from SERS Measurements

**Figure 3.**
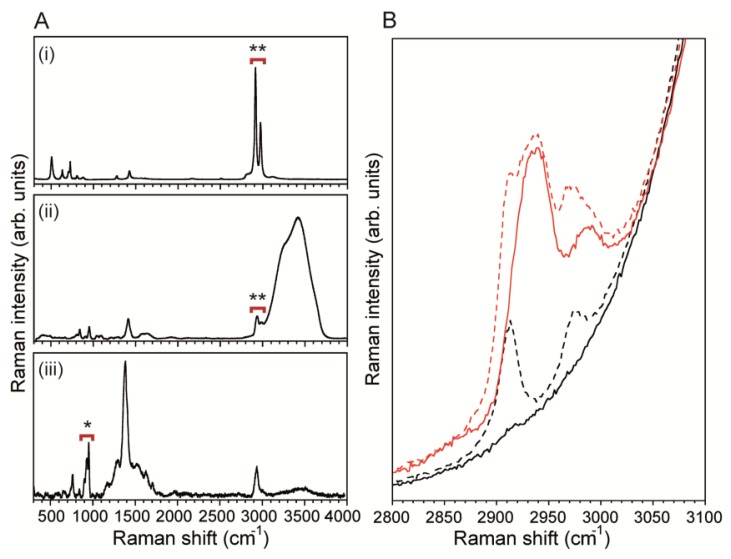
(**A**) Raman spectra of (i) PDMS, (ii) 500 mM sodium citrate solution, and (iii) SER spectra of 5 mM sodium citrate solution over the opaque SERS substrate. The PDMS spectrum in (i) was acquired directly at the surface of PDMS. Sodium citrate spectrum (ii) was acquired in a glass capillary. The region marked by the double star (**) shows the νCH_2_ and νCH_3_ absorption peaks of citrate and PDMS, respectively analysed in (B). The region marked by a single star (*) in (iii) corresponds to the νC-COO stretching region of the sodium citrate which is used for the SERS measurements in the next section. The region marked by the double star (**) shows the νCH_2_ and νCH_3_ absorption peaks of citrate and PDMS, respectively analysed in (B); (**B**) Spectra of 500 mM sodium citrate solution (red) and water (black) as measured with the focal point 50 μm away from the microchannel wall. Solid lines show spectra that were acquired in channels where surfaces are coated by an opaque silver layer and broken lines represent measurements in channels with PDMS-exposed walls.

The MF bioreactor described here was designed to confine and measure the biofilm precursor flow streams along the channel wall where measurements are taken. Due to the close proximity of the channel wall and the analyte molecules, the SERS surface should both enhance sensitivity and block background signals originating from the PDMS channel wall. The latter was critical because PDMS CH_3_ symmetric and asymmetric stretching bands (2,907 cm^−1^ and 2,967 cm^−1^, respectively) [41] overlap with signals from most organic molecules, such as citrate CH_2_ symmetric and asymmetric stretching (2,935 cm^−1^ and 2,986 cm^−1^, respectively) (Figure 3A) [42]. For example, measurements of pure water in the absence of an opaque silver layer at the channel wall revealed νCH_3_ bands from PDMS even in when the confocal measurement point was displaced 50 μm above the channel surface wall (Figure 3B). Even absorption bands from a 500 mM sodium citrate solution were significantly blocked by PDMS νCH_3_ bands. Therefore, SERS surfaces required a continuous opaque metal layer along the channel wall, such that no laser excitation radiation could reach the underlying PDMS. This eliminated interfering PDMS bands, revealing the true spectral characteristics from pure water and the 500 mM sodium citrate solution.

### 3.2. Calibration of the SERS Sensing Surface

The efficacy of our method for achieving good SERS substrates in a microchannel was tested by: (i) the determination of the limit of detection for sodium citrate using a calibration curve; (ii) verification that signals are independent of the flow rate and (iii) determining fast signal response time after a rapid change in analyte concentration.

**Figure 4.**
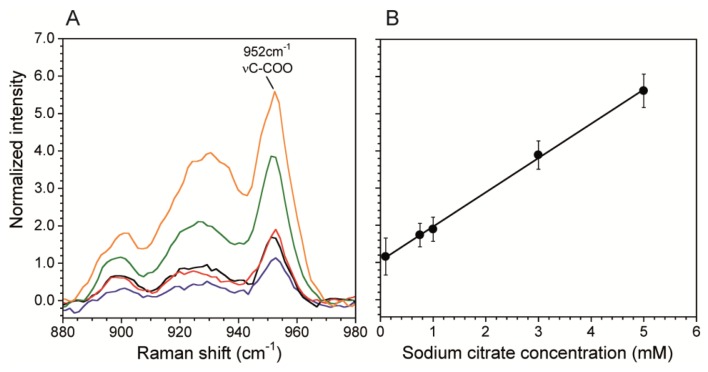
(**A**) Spectra of stationary sodium citrate solutions over silver SERS substrate in the region of C-COO stretching in the concentration range from 0.1 mM to 5 mM acquired using P = 10 mW, *v* = 514.5 nm and t = 60 s. The spectra were normalized with the νO-H band of water at 3,535 cm^−1^. Each spectrum shown is the average of four spectra at same concentration; (**B**) Calibration curve for sodium citrate using the band at 952 cm^−1^. The standard deviations of four measurements at each concentration were used for the error bars.

**Figure 5.**
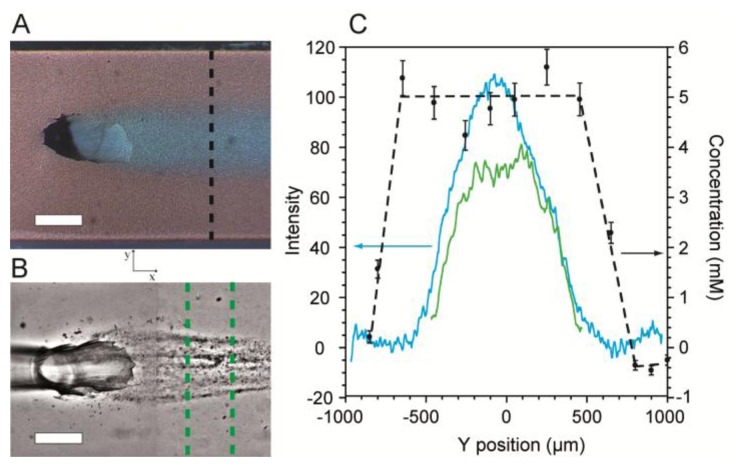
(**A**) An image of the microchannel in the vicinity of the junction with solutions of pure water (red) and 5 mM citrate (blue), respectively. (**B**) Micrograph the flow-templated *Pseudomonas sp.* bacterial biofilm after 48 h growth at 22 °C (with no dyes present in either solution phase) cultivated under the same flow conditions as in (A). (**C**) Cross-sections of the citrate solution and a flow-templated biofilm determined by optical density measurements and SERS. Concentrations profile of the citrate solution (black data points) was measured by SERS along the path marked in (A).The citrate concentration was determined by the conversion of the intensity ratio of νC-COO (952 cm^−1^) and νO-H (3,435 cm^−1^) using a calibration curve (Figure 4B). Error bars were derived from the uncertainty on the linear regression that defined the calibration curve. The black broken line is for eye guidance. The cross-section of the citrate analyte stream from (A) was acquired by image analysis of the blue colour optical density at various paths positions along the length of the channel and averaging their results (solid blue line). The optical density cross-section of a flow-templated biofilm (solid green line) was acquired by averaging data from individual micrographs acquired between 48 and 56 h after inoculationin the region shown in (B). Scale bars in A and B are 500 μm. The flow rates used in A-C were Q_2_ = 0.15 mL/h and Q_1_ = 0.34 mL/h.

A calibration curve correlates Raman scattering intensities to known chemical concentrations in order to quantitatively determine concentrations in unknown solutions. This is particularly important when using SERS surfaces in microchannels because the plasmonic enhancement is very sensitive to the SERS fabrication technique. We generated a calibration curve using the citrate band associated with νC-COO at 952 cm^−1^ (Figure 4A). We chose this band over the *v*CH_2_ because it was stronger and did not have any overlap with *v*O-H band from water, which we used for normalization. Figure 4B shows the normalized intensity of the 952 cm^−1^ band. The curve was collected at the SERS surface prepared as described in Sections 2.2 and 2.3 using data acquisition time *t* = 60 s, laser power *P* = 10 mW. The total flow rate *Q* = 0 mL/hr was used to eliminate any flow induced cooling, thereby demonstrating the benefit having a low P, which resulted in no heat-induced bubble formation. Other measurements using *P* = 100 mW resulted in bubble formation at the channel wall except at high flow rates. In addition, the reduction in energy supplied to the system enabled by SERS, should also strongly reduce other negative effects related to analyte heating, such as photodegradation of analyte molecules and flow distortions due to thermal driven convection cells resulting from heating of the SERS surface at the bottom of the channel. On the other hand, Figure 5B shows the resulting linear calibration curve in the concentration range 0.1 mM and 5 mM. We did not calibrate for lower concentrations because this range was sufficient for the current work, and non-linearities may exist outside of this range [29,43]. Therefore, concentrations below 0.1 mM can only be used for qualitative purposes. For comparison, without the SERS substrate, the limit of detection was 10 mM for acquisitions of *t* = 600 s at laser power of *P* = 250 mW. The enhanced sensitivity in the sub mM range will enable future kinetic studies into biocatalytic degradation of citrate by biofilms. Moreover, the large reduction in acquisition time allows faster generation of spectral images with better spatial resolution.

Next, we verified that the flow rate had no effect on the measurements. In these experiments a 5 mM citrate solution was pumped into the MF bioreactor at flow rates ranging between 0 mL/h and 3 mL/h. Measurements using t = 60 s and P = 10 mW were conducted multiple times at each flow rate. Comparing their averages, it was determined that there was no statistical difference in average signal intensity (data not shown). The signal response in the time following a rapid change in concentration, was achieved by flowing a 5 mM sodium citrate solution at Q_2_ = 0.5 mL/h and then rapidly reducing the flow to Q_2_ = 0 mL/h while increasing the flow of pure water from Q_1_ = 0 mL/h to Q_1_ = 0.5 mL/h. The change in measured signal intensity with time (Figure S3) shows a rapid decrease in peak intensity following an initially slow decrease, which we attribute to residual flow through the system following changes to the pump flow rates.

### 3.3. Spectral Imaging and Validation of Biofilm Precursors

Next, SERS imaging along the cross-section (perpendicular to the direction of flow) of the flow-confined 5 mM citrate stream was conducted to determine its coverage and concentration along the SERS surface. Validation was accomplished using optical microscopy with dyes added for visualization. Figure 5A shows an optical micrograph of a stream of citrate solution (Q_2_ = 0.15 mL/h) flowing along the SERS surface after emerging from the junction between channels in levels 1 and 2. The confinement phase flow rate was Q_1_ = 0.34 mL/h. In order to demonstrate the ability to use the platform to template biofilm growth, we inoculated the MF bioreactor with *Pseudomonas sp.* bacteria and cultured it under a flow-confined citrate growth solution with the same flow conditions used as in Figure 5A. Figure 5B shows a representative high contrast optical micrograph of a spatially confined biofilm after 48 h following inoculation.

The cross-sections of the citrate solution and the flow-templated biofilm from Figure 5A,B were measured quantitatively by a combination of optical density measurements and SERS imaging and plotted in Figure 5C. For SERS images, no dyes were used in order to eliminate extra vibrational absorption bands and to prevent possible fluorescence. Near the channel walls, the measured citrate concentration along the SERS surface was zero, indicating the presence of only the pure water confinement phase. At short distance from either wall, the citrate concentration rapidly increased until it reached a plateau at 5 mM, as expected. The width of the plateau was approximately 1,100 μm. Since SERS efficiency rapidly decreases at distances farther than 3 nm from the metallic surface [44], the plateau width represents the width of the citrate stream along the bottom of the channel. For validation purposes, we measured the optical density from the micrograph along the same path used for SERS. The profile for these measurements had a bell-shaped curve (Figure 5C). The difference in the curves acquired using the two methods arose because transmission optical microscopy produces averaged colour density along the z-direction, whereas SERS only measures the surface concentration. Since the biofilm precursor phase emerges from the bottom of the Level 1, it is widest at the bottom of the channel. Therefore, the location where the optical intensity begins to increase marks the precursor/confinement phase boundary at the SERS surface. This is discussed further in Supplementary Material. The measured width was 1,150 μm, which agrees well with the SERS measurements.

The optical density profile of the templated biofilm from Figure 5B is also plotted in Figure 5C. As expected, biofilm growth was confined to the centre of the channel where the concentration of citrate was non-zero. We note that the biofilm profile followed approximately the same trend as the citrate optical density profiles rather than the citrate SERS profiles. From this information, we deduce that the height of the precursor solution layer near the precursor/confinement solution interface must be too small to provide sufficient nutrients to adsorbed bacteria, thereby preventing growth. This approach can be used in future studies to gain further insights regarding MF studies of biofilms as well as other chemical or biochemical phenomena occurring at microchannel walls.

In addition, the SERS chemical concentration data shows concentration gradients between the zones containing 5 mM and zero citrate concentration. We hypothesize that these are the result of diffusing citrate molecules along the SERS surface between the two solutions. Future experiments are planned to measure concentration gradients and correlate them to molecular diffusion properties along the SERS surface.

## 4. Conclusion and Outlook

A bioreactor with integrated plasmonic surface for SERS has been demonstrated for the spectral and optical imaging of biofilms and their precursor materials. The SERS surface was easily fabricated using equipment available to most research groups and can be applied to large complex channel geometries and is not limited to any microfluidic fabrication material in particular. Enhanced sensitivity was enough to enable sub millimolar sensitivity, fast data acquisition and low laser power. The novel two-level architecture induces sheath flow confinement of the biofilm precursor solution against the plasmonic surface, enabling unobstructed optical and SERS imaging. We observe good complementarity in the flow profile using optical microscopy and SERS imaging. Finally, we successfully flow-templated a *Pseudomonas sp.* bacterial biofilm under dynamic flow conditions. Further optimisations can include exploration of plasma treatment times and conditions, and utilization of laser frequencies that more closely match the plasmonic band of the SERS surface. The microfluidic device and methodology developed opens the way for future *in situ* spectral imaging of biofilms and their biochemical environment under dynamic flow conditions.

## Supplementary Material
