# Supplementary material for: A Microfluidic Bioreactor with in Situ SERS Imaging for the Study of Controlled Flow Patterns of Biofilm Precursor Materials
Source: Sensors (Basel). 2013 Oct 29;13(11):14714–27. doi: 10.3390/s131114714 (PMC3871105; doi:10.3390/s131114714)
Supplement: Supplementary file 1 [file sensors-13-14714-s001.pdf]

OPEN ACCESS

sensors

ISSN 1424-8220

www.mdpi.com/journal/sensors

Supplementary Information

A Microfluidic Bioreactor with *in Situ* SERS Imaging for the Study of Controlled Flow Patterns of Biofilm Precursor Materials. *Sensors* 2013, *13*, 14714-14727

François Paquet-Mercier, Nahid Babaei Aznaveh, Muhammad Safdar and Jesse Greener *

Département de Chimie, Université Laval, 1045 Avenue de la Médecine, Québec, QC G1V 0A6, Canada; E-Mails: francois.paquet-mercier.1@ulaval.ca (F.P.-M.); nahid.babaei-aznaveh.1@ulaval.ca (N.B.A.); muhammad.safdar.1@ulaval.ca (M.S.)

* Author to whom correspondence should be addressed; E-Mail: jesse.greener@chm.ulaval.ca; Tel.: +1-418-656-2131 (ext. 7157); Fax: +1-418-656-7916.

1. Surface Topography

The AFM analysis was conducted for samples after 0, 10 min and 20 min of air plasma treatment.

Figure S1. Roughness factor (Ra) evaluated from AFM images acquired in tapping mode of the silver surface for increasing plasma exposure time. Images were acquired over a surface area of 100 μm^2 at scan rate of 0.25 Hz.

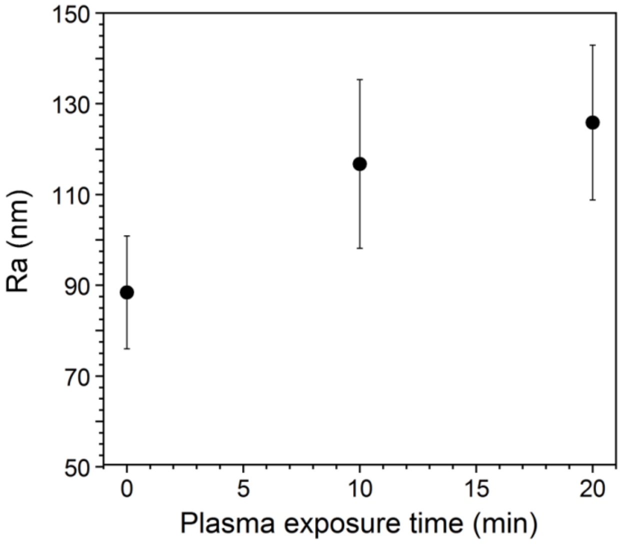

Topography analysis was performed at three different positions on a sample to obtain an average Ra value, which were plotted against plasma exposure times, as shown in Figure S1. An increase of 42% in surface roughness for longer exposure times (20 min) was observed.

2. Absorption Peak Deconvolution and Evolution in Time

UV-Vis diffuse reflectance spectra of the plasma treated silver layers were deconvoluted in order to have more insight into the frequency side of the absorption spectrum and the evolution of the different components with plasma exposure time. Three bands were used for the deconvolution (397 nm, 439 nm and 466 nm). We observe that the intensity of all three bands increases until 16 min of plasma exposure, but then stay stable.

Figure S2. (**A**) Deconvolution spectra of the diffuse reflectance UV-Vis spectra (solid blue line) of the silver surface after 10 min air plasma exposure (600 mTorr, 29.6 W). The first, second and third bands used for the deconvolution are centered at 397 nm, 439 nm and 466 nm and are displayed in orange, green and black broken lines, respectively, with their sum shown as a broken red line; (**B**) Intensity of the bands at 397 nm, 439 nm and 466 nm (orange dots, green squares and black triangles, respectively) taken from the deconvolution spectra for different plasma exposure times.

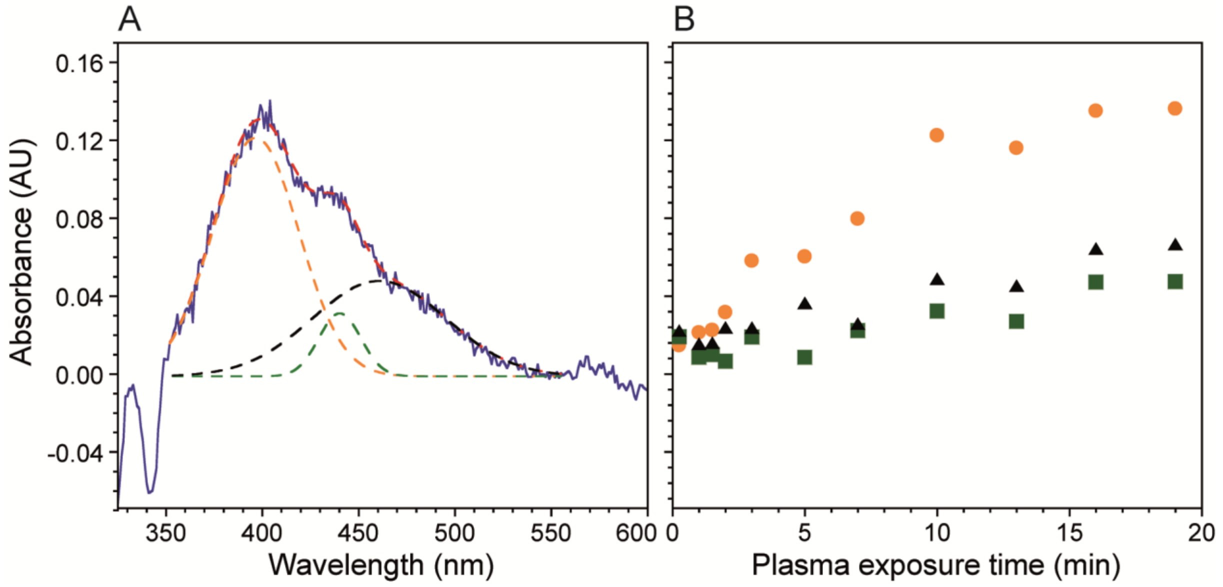

3. Time Response of SERS Measurements

In order to verify that fast changes in analyte concentration can be tracked by SERS, we flowed a 5 mM sodium citrate solution through Inlet 2 at Q_2 = 0.5 mL/h, until the signal reached a maximum and stabilized. Following this, we set Q_2 = 0 mL/h and increased the flow of pure water in Inlet 1 from Q_1 = 0 mL/h to Q_1 = 0.5 mL/h. Spectra were continuously acquired every 15 s starting when the flow rates were changed. We used the intensity of the citrate νC-COO band at 952 cm^{-1} to follow the decrease in measured concentration as water replaced the citrate solution at the SERS surface in the channel (Figure S3). We observed a slow initial decrease from 0 min to 2 min and 35 s, which we

attribute to the residual flow in the system following the change in the flow rates due to pressure buildups in the syringe pumps. After residual pressure was eliminated, we observed a rapid decrease in the intensity to 0 mM from 2 min 35 s to 2 min 48 s, where the measurement stayed constant for the duration of the experiment. This demonstrates that the SERS surface itself is capable of rapid responses to changes in concentration.

Figure S3. Evolution of the SERS intensity of the 952 cm^{-1} band of sodium citrate after replacement by water. Initially, a 5 mM sodium citrate solution was flown through Inlet 2 at 0.5 mL/h. The flow in Inlet 2 was then set to 0 mL/h while increasing the flow of pure water in Inlet 1 at 0.5 mL/h. Spectra were continuously acquired every 15 s. Acquisition was conducted using P = 10 mW, and v = 514.5 nm. The time, t, indicates the overall time for the system to respond.

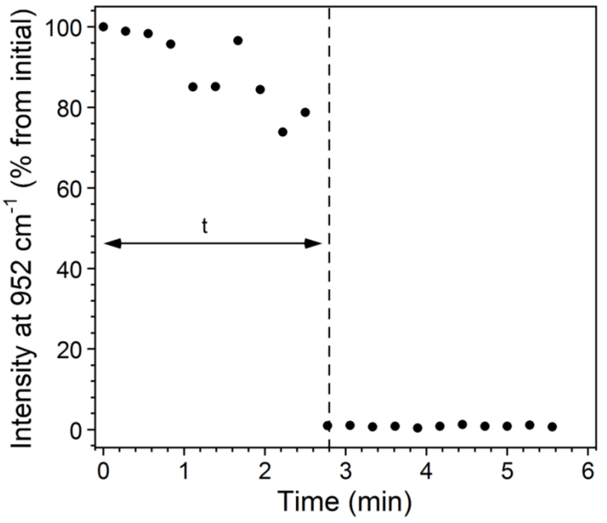

4. Optical Density Measurements

Flow profiles measurements were collected from optical micrographs to validate Raman spectral images. Blue and red dyes were added to analyte streams and confinement streams, respectively, and the resulting optical images were separated into two images according to their blue and red colour content (Figure S4A). Using freeware image analysis software (ImageJ v1.47), baseline corrections were conducted on both images, to account for illumination gradients in the original image, followed by measurements of pixel brightness intensity along the linear path, perpendicular to the direction of flow. The results show two peaks, which rise/fall in opposition to the other (Figure S4B). Since optical images were collected in transmission mode, each pixel represents the z-averaged blue/red colour (*i.e.*, the average colour along the vertical direction at each y-position along the profile path). Due to the laminar flow properties within microchannels, streams are actually stratified, such that for some points along the analysis path, there are regions near the top and bottom of the channels where streams are comprised of different liquid streams.

Figure S4. (**A**) The colour optical micrograph collected in the transmission mode for a typical flow confined liquid system with flow rates Q_1 = 0.34 mL/h and Q_2 = 0.14 mL/h. The arrow shows the analysis path followed to generate colour density cross-sections. (**B**) Colour density cross-sections from optical micrograph in (A) showing the cross-section of the flow of the biofilm precursor stream (blue) by a confining stream (red). The profile is marked by regions where the volume of liquid is represented by pixels probing only confining liquid (red stars) or only analyte liquid (blue stars). At other locations in the cross-section the colour density is comprised of stratified red and blue streams marked by a two-colour star. Scale bar in (A) is 500 μm

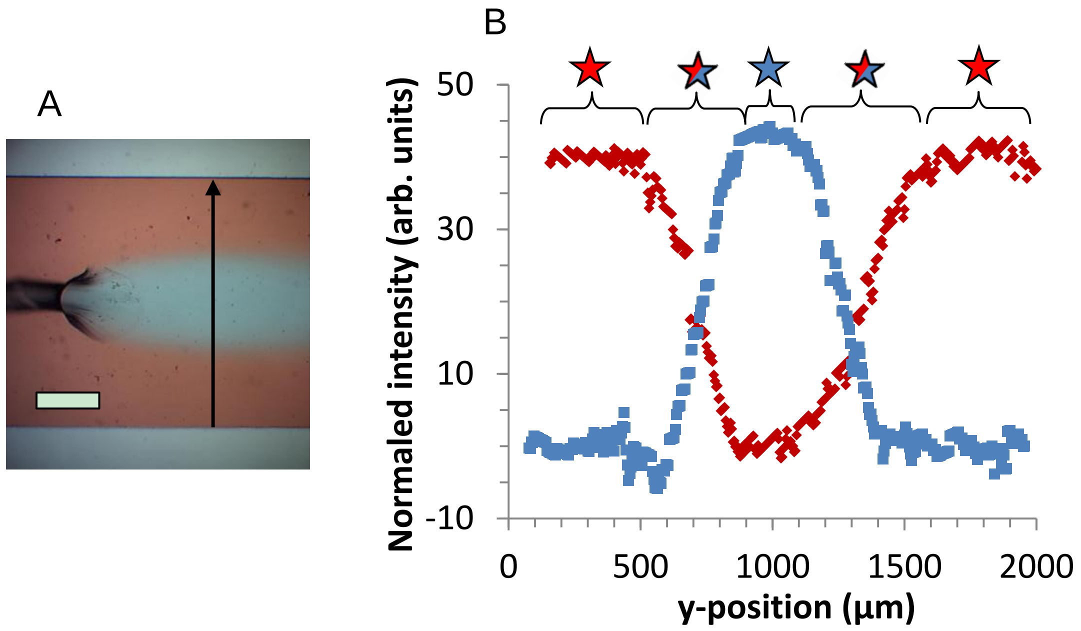

5. Biological Material Preparation

Colonies of *Pseudomonas sp* strain CTO7 were cultured at 24 °C overnight on a AB nutrient Petri dish. For fluorescence microscopy we used bacteria that had green fluorescent protein (GFP) inserted into the bacterial chromosome as a fluorescent label. Pre-culture used as inoculum for bacteria seeding were obtained by scraping bacteria colonies from the surface of AB plate and transferred to a tube containing 5 mL, AB nutrient 5 mM. The tube was incubated for 18 h on the shaker at 30 °C, at 300 rpm. The tube connectors and the microfluidic channels were disinfected by flowing 70% ethanol for 2 h and then sterile distilled water (autoclaved) for 1 hour sequentially at 5 mL/h.

Both bacteria pre-culture and a nutrient bath were injected through a T-connection were connected to Inlet 2 in the second level. To avoid mixing the two solutions, the valves were used before entering the tubes to T-connection. After sterilizing, the flow of water and nutrient passed through the channels for 30 min. The valve was closed to nutrient flow and open to bacteria flow during inoculation. Inoculation was performed for 45 min under flow. After inoculation was finished, the valve was opened to the biofilm growth solution and closed to the inoculant liquid so that the seeded bacteria could be cultured in a growth solution. All the growth solutions were sterilized prior to injection in the microfluidic bioreactor.

6. Biofilm Growth on Silver and Gold Layers

Future experiments will use Raman spectral imaging to monitor the growth of biofilms against a SERS surface. Since silver is typically considered an antimicrobial agent, biofilm growth was attempted against a silver layer after electroless deposition in the channel. Two similar tests were undertaken, one on silver, one on gold. In these tests, the channel was inoculated with a flow of planktonic bacteria in growth solution through Inlet 2 (Figure 1), followed by a flow of citrate growth solution (5 mM). Pure water was flowed though Inlet 1 to selectively inhibit biofilm growth. The flow rates of the Inlet 1 and Inlet 2 liquids were such that inoculation and biofilm growth was confined to the three metalized walls, but was inhibited at the glass surface. As seen from Figure S5, biofilm growth occurred on both silver and gold metal layers. Evidently, the biofilm's protective matrix that surrounds bacteria is sufficient to mask the antimicrobial effects of the silver surface.

Figure S5. Fluorescence microscope images of biofilms containing GFP bacteria in microchannels containing a silver layer. (**A**) Microscope image with focal point adjusted to the channel bottom shows in-focus biofilm attached to the silver layer. Small fractures and defects in the silver layer can be seen through the biofilm. (**B**) Same field of view shown in (A), but with focal plane moved to the glass slide. Out of focus biofilm shows that biofilm is not touching the glass and therefore the GFP signal is coming from the bottom of the channel. (**C**) Shows a similar image to (A), but on electrolessly deposited gold. (**D**) Shows a similar image to (B) for the gold coated microchannel. In (B) and (D) a small amount of biofilm is in focus at the wall of the microchannel, demonstrating that some biofilm has reached the wall and climbed in the z-direction to reach the focal plan at or close to the glass slide. A reference image for each channel was acquired with no GFP biofilm present, showing that the GFP bacteria are the only source of fluorescent light. Scale bars are 20 μm.

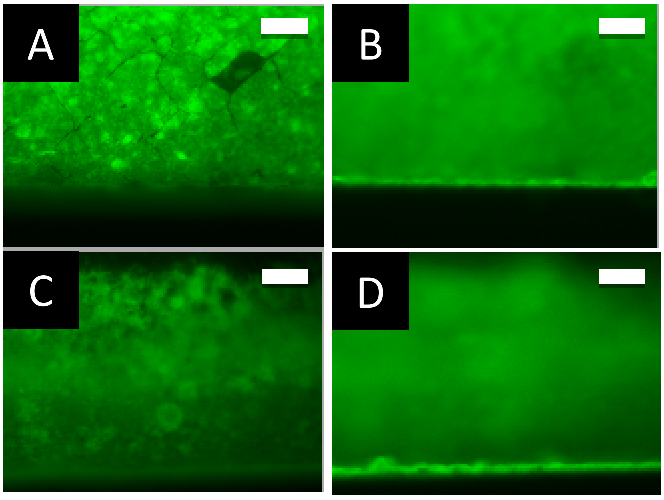

© 2013 by the authors; licensee MDPI, Basel, Switzerland. This article is an open access article distributed under the terms and conditions of the Creative Commons Attribution license (http://creativecommons.org/licenses/by/3.0/).
